# 3D smooth path planning of AUV based on improved ant colony optimization considering heading switching pressure

**DOI:** 10.1038/s41598-023-39346-5

**Published:** 2023-07-31

**Authors:** Ronghua Meng, Aiwen Sun, Zhengjia Wu, Xuan Du, Yongdong Meng

**Affiliations:** 1 Hubei Key Laboratory of Construction and Management in Hydropower Engineering, China Three Gorges University, Yichang, 443002 Hubei China; 2 Hubei Key Laboratory of Hydroelectric Machinery Design and Maintenance, China Three Gorges University, Yichang, 443002 Hubei China; 3 Intelligent Manufacturing Innovation Technology Center, China Three Gorges University, Yichang, 443002 Hubei China; 4 School of Management, Jinan University, Guangzhou, 510632 Guangdong China

**Keywords:** Engineering, Mathematics and computing, Applied mathematics

## Abstract

A smooth and secure spatial path planning algorithm that integrates the improved ant colony optimization with the corrective connected spatial search strategy is proposed, aiming at heavy heading switching pressure of autonomous underwater vehicles sailing in complex marine environment. On the one hand, to overcome the low-dimensional search domain and inaccurate spatial communication information in traditional spatial path planning, the spatial connectivity adjacency domain search strategy is designed based on grid environment model. On the other hand, to alleviate heading switching pressure due to large path steering angles and redundant path turning points, the heuristic functions and pheromone update criterion based on ant colony optimization are introduced to improve the solution quality of smooth paths. The simulation results show that the space search strategy can improve the success probability of safe path planning without reducing the scope of explorable free space. Additionally, the simulations demonstrate that the improved ant colony optimization using the spatial search strategy can guarantee the shortest path with lowest tortuous degree and fewest turning times in the same grid environment.

## Introduction

Autonomous underwater vehicle (AUV) is a commonly used underwater vehicle for ocean exploration^1^. The energy storage of AUV limits its working capacity, and the complex underwater water environment proposes a great threat to the navigation safety of AUV. Path planning technology can ensure the safe navigation of AUV, optimize the path and improve its work efficiency, which is one of the key technologies of intelligent control^2^**.** Path planning designs mathematical models and path planning strategies adapted to each optimization index (path security^3–5^, path length^6,7^, path smoothness^8,9^) based on environmental map information, and apply intelligent algorithms to apply solutions. Two main factors that affect the quality of the solution results: the fineness of the environment model and the strength and weakness of the algorithm itself^10^.

Path planning methods can be applied in both spatial and planar domains. Traditional environment modeling methods include grid method^11^, topology method^12^ and visibility graph method^13^ et.al. The spatial environment model can be constructed by superimposing the dimensions of the planar model. In order to balance the computational pressure caused by the dimensionality expansion of the environment model, the path search dimension has been reduced to planar in some spatial path planning studies. This approach does not sufficiently exploit the spatial environment information to achieve substantial spatial path planning. For example, Bae H^14^ proposed dimensional transformation method, which generates paths in 2D planes from start points to target points, and then converts them into 3D paths.

The AUV path planning algorithms mainly include: sampling-based method (RRT^15^), mathematical optimization algorithm based on polynomial (Dubins curve^16^, Bezier curve^17^), algorithm based on geometric model search (Dijkstra^18^, A*^19^), algorithm based on biological intelligence (PSO^20^, ACO^21^, GNN^22^, GA^23^, APF^24^). Willners^25^ proposes that the maintenance of the hybrid HA* path extender can effectively search and shorten the planning time, merely the overall adaptability should be improved. Hussein M^26^ proposes the RRT * algorithm for directivity exploration, with computational complexity and the unsmooth path. Fan^27^ combines RHG and introduces distance correction factor into APF to remedy the inherent defects that local minimum value and target cannot be obtained. Wang^28^ introduced safety-value heuristics to improve the ant colony optimization and applied a 3D path lock-free mechanism, which resulted in smooth paths and saved computational power. Ant colony optimization (ACO) has a strong ability to search for a single shortest path objective, outperforming other intelligent algorithms. However, the ability of the ACO is inefficient to balance the path length and the degree of tortuousness. So, it will be improved to solve this path planning issue in this paper.

Obstructions are spatially densely distributed in complex marine environments. The AUV must avoid obstacles in the environment to reach the target location safely. The tortuous path to avoid underwater obstacles accentuates the heading switching pressure on the AUV and reduces the efficiency of subsea operations. Aiming at the heavy heading switching pressure caused by the AUV's tortuous path. An organic combination of search strategy and intelligent algorithm is used to investigate this issue in this paper. Considering the accurate application of spatial environment information, a modified connected spatial search strategy based on grid method is proposed. Improving the dimensionality of the search domain and avoiding known static obstacles for secure spatial path planning. Integrating with the spatial search strategy, a path planning algorithm is optimized based on ant colony optimization. Adding the heuristic function and refining the pheromone update mechanism of ACO, can optimize the path length and path tortuosity influenced by obstacle avoidance.

## Problem model

### Problem description

This paper studies the issue of the shortest and smoothest point-to-point path planning for AUV in a spatial environment with complex distributed obstacles.

Assumptions: (1) the environmental information is on known; (2) the AUV operating with sufficient energy; (3) the start location and target location of AUV are determined.

### Environmental model

Applying the grid method to environment modeling, the principle is to discretize the whole working environment into a non-overlapping adjacent grid set domain by grids with appropriate granularity. The environmental information of each grid contains the positioning information and traffic state of the corresponding actual space^29^. Focusing on the dimensionality of the AUV operating environment, using a unify-size cubic gird body to discrete environment space. The new grid granularity partitioning principle: the AUV solid structure is upgraded to a cube, and the longest edge length of the cube *l* is set to be the size of the grid body l*l*l, the centroid coordinates of a grid represent its spatial location. Setting the activity value of obstacle grid as *Inf* and the activity value of free grid as 0. The environment model formed by discretizing the 3D environment space B*E*F is the spatial grid set domain b*e*f. *B*, *E* and *F* are the length, width and height parameters of the 3D environment space, respectively. *b*, *e* and *f* are the row, column and layer parameters of the spatial grid set domain, respectively. *ceil* in formula (1) is the integer up operation.

1 $$\left\{ \begin{gathered} b = ceil(B/l) \hfill \\ e = ceil(E/l) \hfill \\ f = ceil(F/l) \hfill \\ \end{gathered} \right.$$

**Figure 1:**
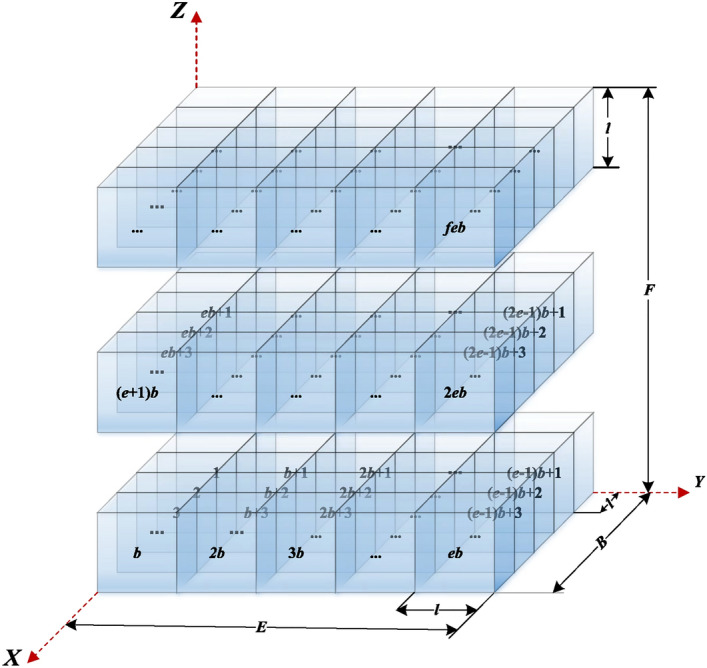
Environmental model characterization.

The representation of the environment model spatially using the grid method is shown in figure 1.

Summarizing the distributed condition of the obstacles constitutes the grid map's the environment information matrix, which holds the traffic situation of the location. Let the grid's serial number equal to the index of the environment information matrix, facilitating the computer calculation. The conversion between centroid coordinates $(x_{i} ,y_{i} ,z_{i} )$ and serial number $i$ of the grid body such as formula (2), $Mod( \cdot )$ is the remainder operation, $Floor( \cdot )$ is the round-down operation.

2 $$\left\{ \begin{gathered} x_{i} = Mod(i - Floor(i/eb) \cdot eb - 1,b) + 0.5 \hfill \\ y_{i} = Floor((i - Floor(i/eb) \cdot eb - 1)/b) \hfill \\ z_{i} = Floor(i/eb) + 0.5 \hfill \\ \end{gathered} \right. + 0.5$$

### Mathematical model

3 $$Rout^{k} = \{ g_{1} ,g_{2} ,g_{3} ,...,g_{n - 1} ,g_{n} \}$$

Path result represents as formula (3), $k$ is the path ordinal number, $g_{i}$ is the grid body serial number. A successful path is formed by starting point $S$, target point $E$, and $n - 2$ free grid bodies conforming in order.

4 $$f_{1} = \min PL$$

5 $$f_{2} = \min P\theta$$

6 $$d_{{g_{i - 1} ,g_{i} }} = \sqrt {(x_{{g_{i - 1} }} - x_{{g_{i} }} )^{2} + (y_{{g_{i - 1} }} - y_{{g_{i} }} )^{2} + (z_{{g_{i - 1} }} - z_{{g_{i} }} )^{2} }$$

7 $$PL^{k} = \sum\limits_{i = 2}^{n} {d_{{g_{i - 1} ,g_{i} }} }$$

8 $$\theta_{{g_{i - 1} ,g_{i} }} = \cos^{ - 1} (\frac{{\left( {x_{{g_{i - 2} }} - x_{{g_{i - 1} }} } \right) \cdot \left( {x_{{g_{i - 1} }} - x_{{g_{i} }} } \right) + \left( {y_{{g_{i - 2} }} - y_{{g_{i - 1} }} } \right) \cdot \left( {y_{{g_{i - 1} }} - y_{{g_{i} }} } \right) + \left( {z_{{g_{i - 2} }} - z_{{g_{i - 1} }} } \right) \cdot \left( {z_{{g_{i - 1} }} - z_{{g_{i} }} } \right)}}{{\sqrt {(x_{{g_{i - 2} }} - x_{{g_{i - 1} }} )^{2} + (y_{{g_{i - 2} }} - y_{{g_{i - 1} }} )^{2} + (z_{{g_{i - 2} }} - z_{{g_{i - 1} }} )^{2} } \cdot \sqrt {(x_{{g_{i - 1} }} - x_{{g_{i} }} )^{2} + (y_{{g_{i - 1} }} - y_{{g_{i} }} )^{2} + (z_{{g_{i - 1} }} - z_{{g_{i} }} )^{2} } }})$$

9 $$P\theta^{k} = \sum\limits_{i = 2}^{n} {\theta_{{g_{i - 1} ,g_{i} }} }$$

Formula (4) is the first objective function of minimizing the path length. Path length *PL* is calculated by formula (6) and (7). $d_{{g_{i - 1} ,g_{i} }}$ is the linear distance from $g_{i - 1}$ to $g_{i}$. Formula (5) is the second objective function of minimizing the path tortuosity. Path tortuosity $P\theta$ is calculated by formula (8) and (9). $\theta_{{g_{i - 1} ,g_{i} }}$ is the path turning degree from $g_{i - 1}$ to $g_{i}$,$\theta_{{g_{i - 1} ,g_{i} }} \in [0,180][0,180]$.

10 $$2 - P_{1}^{k} - P_{2}^{k} = 0$$

11 $$P_{1}^{k} = \left\{ \begin{gathered} 1,\quad Rout^{k} (last) = E \hfill \\ 0,else \hfill \\ \end{gathered} \right.$$

12 $$P_{2}^{k} = \prod\limits_{i = 2}^{i = n} {N(g_{i - 1} ,g_{i} )}$$

13 $$N(g_{i - 1} ,g_{i} ) = \left\{ \begin{gathered} 1,\quad g_{i - 1} {\text{ to }}g_{i} {\text{ available}} \hfill \\ 0,\quad g_{i - 1} {\text{ to }}g_{i} {\text{ unavailable}} \hfill \\ \end{gathered} \right.$$

Formula (10) is the constraint of path to safely arrive the target location. $P_{1}^{k}$ in formula (11) is the arrival path constraint. If the path can reach the target location $P_{1}^{k} = 1$, then the arrival path constraint is satisfied. $P_{2}^{k}$ in formula (12) is the safe path constraint. $N(g_{i - 1} ,g_{i} )$ represents the connection relation between adjacent grids, as formula (13). When the connection relation is correct, $N(g_{i - 1} ,g_{i} ) = 1$. If all adjacent grids are connected correctly in the path result $P_{2}^{k} = 1$, then the safe path constraint is satisfied.

14 $$K(x^{k} ) = \frac{{x^{k} - x_{\min } }}{{x_{\max } - x_{\min } }}$$

The Min Max normalization method is used to normalize the two indicators $PL$ and $P\theta$, as formula (14).

15 $$P^{k} = (2 - P_{1}^{k} - P_{2}^{k} ) \times 1000$$

16 $$fit^{k} = P^{k} + \lambda \cdot K(PL^{k} ) + (1 - \lambda ) \cdot K(P\theta^{k} )$$

$P^{k}$ in formula (15) is comprehensive restraint punishment. As formula (16), $fit^{k}$ is bi-objective comprehensive fitness, the smaller $fit^{k}$, the higher the superiority of the path.

## Spatial search strategy

**Figure 2:**
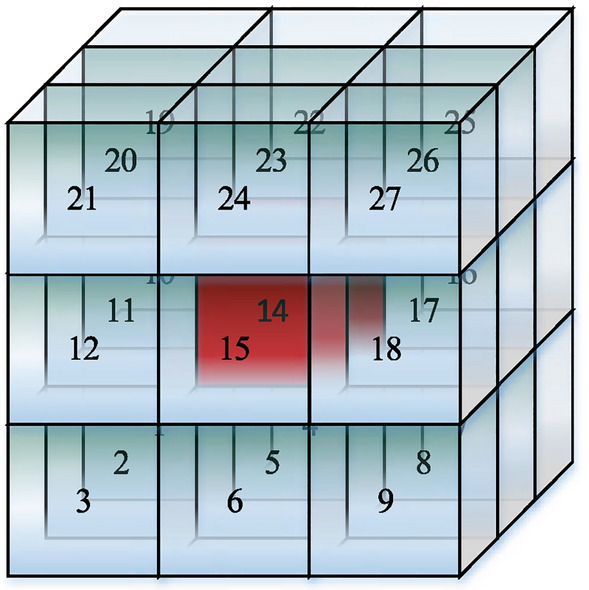
Adjacency search domain.

With the grid as the center, aggregating adjacent free grids as the path space search domain. A grid can have at most 26 directional path choices spatially, as shown in Fig. 2.

**Figure 3:**
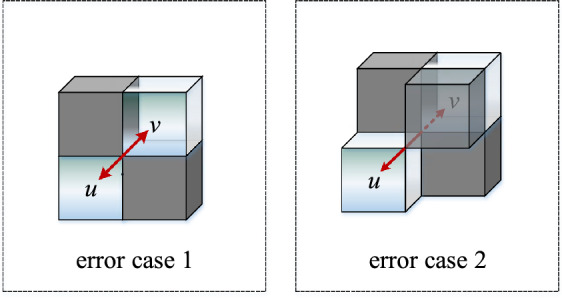
Crossing-type connectivity error.

**Figure 4:**
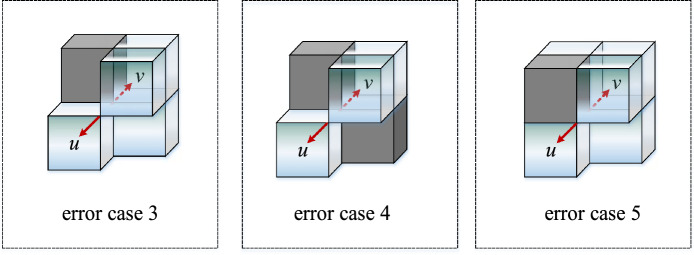
Touch-type Unicom error.

The traditional method of screening grid activity value constructing the spatial search domain, the path may have through-type and touch-type connectivity errors, as shown in Figs. 3 and 4. To illustrate the types of connectivity errors, Figs. 3 and 4 are fixed viewing angles. The structures shown in Figs. 3 and 4 can be rotated in three dimensions to derive all spatial connectivity errors.

In view of the above connectivity errors, this paper proposes a search strategy of spatial connectivity adjacency domain search strategy (SCADSS) to correct the connectivity relation in the search domain. SCADSS constructs the spatial search domain by centering on a free grid body then expanding the correct connectivity adjacency free grid body with three adjacency expansion rules. The three adjacency expansion rules: face adjacency expansion rule (FAER), edge adjacency expansion rule (EAER), point adjacency expansion rule (PAER). Each adjacency expansion rule contains two criterions: the adjacency location criterion and the adjacency connectivity criterion. Applying SCADSS, the correct connectivity relation can ensure secure spatial path planning. The three adjacency expansion rules are as follows.

Setting: central free grid body as $u(x_{u} ,y_{u} ,z_{u} )$, unknown connectivity free grid body as $v(x_{v} ,y_{v} ,z_{v} )$, auxiliary judgment grid body as $w(x_{w} ,y_{w} ,z_{w} )$. Recording : the number of auxiliary judgment free grid bodies for FAER as $Number_{F - w} (u,v)$, the number of auxiliary judgment free grid bodies for EAER as $Number_{E - w} (u,v)$, the number of auxiliary judgment free grid bodies for PAER as $Number_{P - w} (u,v)$. $N(u,v)$ is defined in Formula (13), holding the correct connectivity relation between the two grid bodies.

### Face adjacency expansion rule

- Adjacency location criterion of FAER:
  If the free grid body $v$ and free grid body $u$ satisfy $|x_{u} - x_{v} | + |y_{u} - y_{v} | + |z_{u} - z_{v} | = 1$, the free grid body $v$ is at the face adjacency location of grid body $u$.
- Adjacency connectivity criterion of FAER:
  The free grid body $v$ is at the face adjacency location of grid body $u$.When $field(u) = 0$ and $field(v) = 0$, an auxiliary judgment grid body $w$ exists, the grid body $w$ is the grid body $u$ itself. If $Number_{F - w} (u,v) = 1$, then $N(u,v) = 1$, the correct connectivity relation between grid body $v$ and grid body $u$ is established.

**Figure 5:**
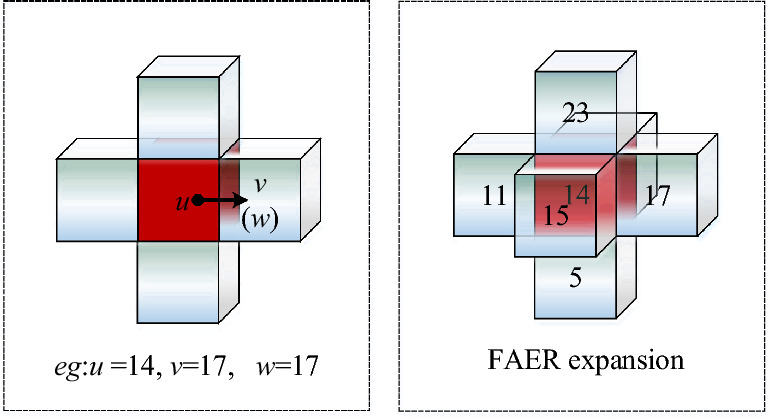
Face adjacency expansion.

In Figure 5, the left figure is the interpretation of FAER and the right figure is the optimal result for FAER. With red grid body as the center grid, the application FAER can expand the path search domain of 6 directions connected correctly at most.

### Edge adjacency extension rule

- Adjacency location criterion of EAER:
  $${\text{condition1}}:\;|x_{u} - x_{v} | = 1 \wedge |y_{u} - y_{v} | = 1 \wedge |z_{u} - z_{v} | = 0$$
  $${\text{condition2}}:|x_{u} - x_{v} | = 1 \wedge |y_{u} - y_{v} | = 0 \wedge |z_{u} - z_{v} | = 1$$
  $${\text{condition3}}:|x_{u} - x_{v} | = 0 \wedge |y_{u} - y_{v} | = 1 \wedge |z_{u} - z_{v} | = 1$$
  If the free grid body $v$ and free grid body $u$ satisfy one of above three conditions, the free grid body $v$ is at the edge adjacency location of grid body $u$.
- Adjacency connectivity criterion of EAER:
  The free grid body $v$ is at the edge adjacency location of free grid body $u$. When the grid body $w$ is both at the face adjacency location of the grid body $u$ and the grid body $v$, an auxiliary judgment grid body $w$ exists. If $Number_{E - w} (u,v) = 2$, then $N(u,v) = 1$, the correct connectivity relation between grid body $v$ and grid body $u$ is established.

**Figure 6:**
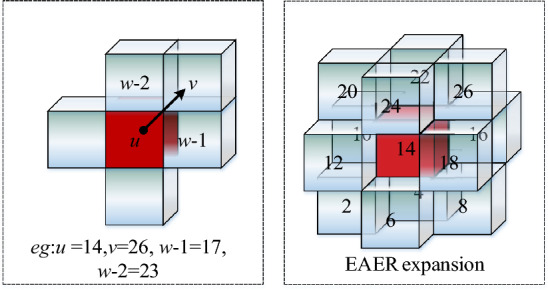
Edge adjacency expansion.

In Figure 6, the left figure is the interpretation of EAER and the right figure is the optimal result for EAER. With red grid body as the center grid, the application EAER can expand the path search domain of 12 directions connected correctly at most.

### Point adjacency extension rule

- Adjacency location criterion of PAER:
  If free grid body $v$ and free grid body $u$ satisfy $\left| {x_{u} - x_{v} } \right| = 1 \wedge \left| {y_{u} - y_{v} } \right| = 1 \wedge \left| {z_{u} - z_{v} } \right| = 1$, the free grid body $v$ is at the point adjacency location of grid body $u$.
- Adjacency connectivity criterion of PAER:
  The free grid body $v$ is at the point adjacency location of free grid body $u$. When the grid body $w$ is both at the edge adjacency location of the grid body $u$ and the grid body $v$, an auxiliary judgment grid body $w$ exists. If $Number_{P - w} (u,v) = 3$, then $N(u,v) = 1$, the correct connectivity relation between grid body $v$ and grid body $u$ is established.

**Figure 7:**
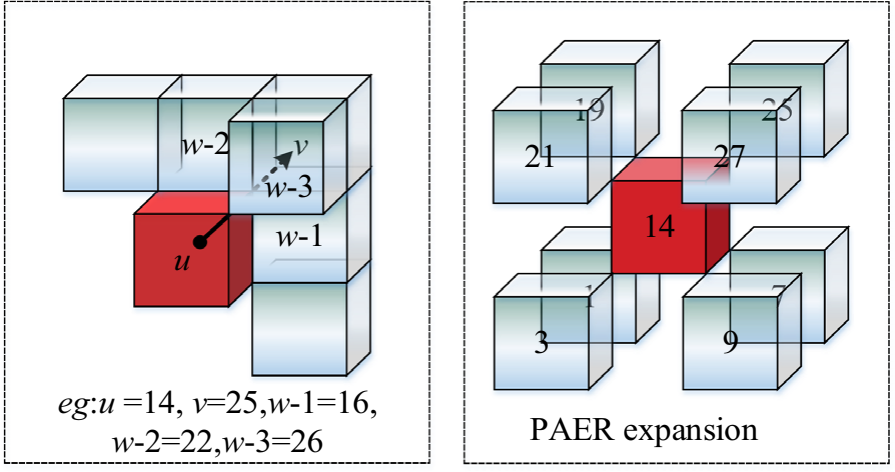
Point adjacency expansion.

In Figure 7, the left figure is the interpretation of PAER and the right figure is the optimal result for PAER. With red grid body as the center grid, the application PAER can expand the path search domain of 8 directions connected correctly at most.

Through the three rules of SCADSS, all the grid bodies $v$ correctly connecting with grid bodies $u$ could be expanded. The grid bodies $v$ form set $A_{u}$. $A_{u}$ is the space search domain that ensures path security by modifying connective relation.

## AUV autonomous path planning algorithm

The AUV updates the current position $i$ in real time from the start location, and picks the next better position $j$ in the search domain $A_{i}$ of $i$. The search operation is repeated until the search domain is empty or the target location is reached. Heuristics are designed to adapt the ACO algorithm to the optimization requirements. The pheromone update mechanism has been optimized to improve the convergence rate of the algorithm.

### Improved distance heuristic function

The Euclidean distance $d_{jE}$ from the search position to the target point is introduced to enhance the optimal path guidance of the distance heuristic function $\eta{\prime} (t)$, as formula (17). To avoid the problem that the traditional ACO algorithm only relies on path visibility $d_{ij}$ to produce a large number of poor initial solutions in the initial operation.

17 $$\eta{\prime} (t) = \frac{1}{{\left( {d_{ij} } \right)^{2} + \left( {d_{jE} } \right)^{2} + \left( {3 \cdot d_{ij} \cdot d_{jE} } \right)}}$$

### Local turning heuristic function

The local path turning angles that may be generated by the path heading change include:$0^{ \circ }$, $1^{ \circ }$, $36^{ \circ }$, $46^{ \circ }$, $55^{ \circ }$, $61^{ \circ }$, $71^{ \circ }$, $90^{ \circ }$, $110^{ \circ }$, $120^{ \circ }$, $126^{ \circ }$, $145^{ \circ }$. In order to reach the shortest path, the same path point cannot be selected twice when searching a path, so there is no possibility of $180^{ \circ }$. $0^{ \circ }$ means heading straightly. A larger value of $\theta_{ij}$ increases the space and energy burden of AUV path deflection. Consider the burden of AUV deflection course, especially the safety of turning operation while avoiding obstacles. The local turning angle heuristic function $\mu (t)$ is designed to measure the rationality of path direction selection, as the formula 18.

18 $$\mu (t) = e^{{\frac{{\cos (\theta_{ij} ) + 1}}{2}}}$$

### Global comprehensive guidance heuristic function

**Figure 8:**
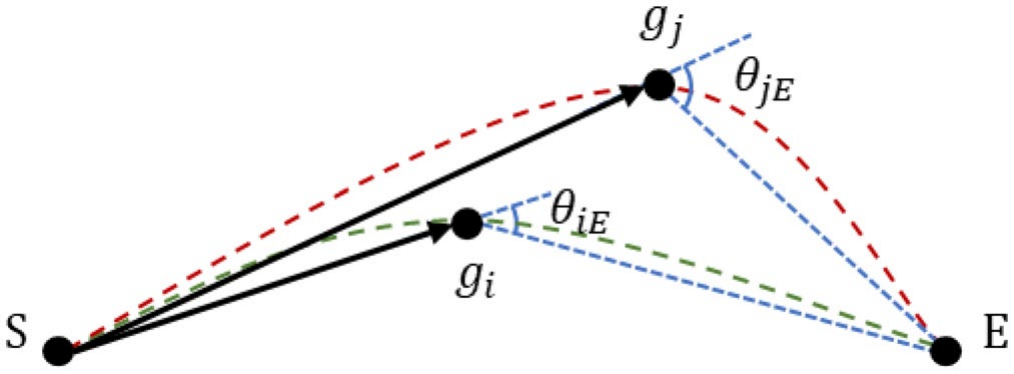
comprehensive guidance angle.

A global comprehensive guidance heuristic function $\varphi (t)$ is designed, as shown in formula (19), to evaluate the influence of location selection on the overall path tortuosity. Avoid path searching generates the shortest path and the gentlest path. The trend of comprehensive guidance path exploration tends to the optimal solution of dual objective path planning problem. The integrated guidance angle $\theta_{jE}$ is shown in Fig. 8.

19 $$\varphi (t) = e^{{\frac{{\cos (\theta _{{jE}} ) + 1}}{6}}}$$

### Path selection probability

Based on the heuristic functions designed, the improved path location selection probability $P_{ij}^{k} (t)$ as shown in formula (20).

20 $$P_{ij}^{k} (t)\left\{ \begin{gathered} \frac{{[\tau_{ij} (t)]^{\alpha } \cdot [\eta_{ij}{\prime} (t)]^{\beta } \cdot [\mu_{ij} (t)]^{\gamma } \cdot [\varphi_{ij} (t)]^{\varepsilon } }}{{\sum\limits_{{u \in A_{i}^{allowed} }} {[\tau_{iu} (t)]^{\alpha } \cdot [\eta_{iu}{\prime} (t)]^{\beta } \cdot [\mu_{iu} (t)]^{\gamma } \cdot [\varphi_{iu} (t)]^{\varepsilon } } }},j \in A_{i}^{allowed} \hfill \\ 0,j \notin A_{i}^{allowed} \hfill \\ \end{gathered} \right.$$

### Dynamic adjustment strategy of pheromone volatilization factor

ACO algorithm iterates the optimal solution through pheromone. Pheromone volatile factor $\rho$ directly affect the convergence of the algorithm. In the early stage of iteration, $\rho$ should be large to attenuate the poor solutions. In the middle stage of iteration, $\rho$ should be moderate to accelerate the screening of better solutions. In the later stage of iteration, $\rho$ should be smaller to urge global convergence. The scaling property of the function $f(x)$ as formula (21) satisfies the above requirements and ensures the $\rho$ value is not too small. The dynamic regulation strategy of pheromone volatile factors as shown in formula (22). $f_{mid}^{t}$ is the average fitness of the result of every iteration. $f_{best}^{t}$ is the optimal fitness value of each iteration.$\rho_{f} (t)$ is dynamically adjusted according to the solving quality of each generation iteration to avoid the interference of inferior solution and improve the algorithm convergence speed. $a$ is a constant keeping $\rho_{f} (t) \in (0,1)$.

21 $$f(x) = \frac{1}{2\sqrt \pi }e^{{ - \frac{{x^{2} }}{2}}}$$

22 $$\rho_{f} (t) = a \cdot \sqrt {{\raise0.7ex\hbox{${f_{mid}^{t} }$} \!\mathord{\left/ {\vphantom {{f_{mid}^{t} } {f_{best}^{t} }}}\right.\kern-0pt} \!\lower0.7ex\hbox{${f_{best}^{t} }$}}}$$

23 $$\rho (t + 1) = \frac{1}{2\sqrt \pi }e^{{ - \frac{{(\rho (t) - \rho_{f} (t))^{2} }}{2}}}$$

### Comprehensive pheromone updating strategy

In order to show the effect of dual-objective planning (shortest path length path and least path tortuosity) in pheromone, the improved integrated pheromone increment is shown in formula (24)–(25). $fit_{t}^{k}$ is the fitness.$\omega$ is the fitness enhancement factor. $\Delta \tau_{ij}^{k}$ represents the pheromone released on grids.

24 $$\tau_{ij} (t + 1) = (1 - \rho (t + 1)) \cdot \tau_{ij} (t) + \sum\limits_{k = 1}^{K} {\Delta \tau_{ij}^{k} (t)}$$

25 $$\Delta \tau_{ij}^{k} = \left\{ \begin{gathered} \left( {\omega fit_{t}^{k} } \right)^{ - 1} ,ant^{k} traverses(i,j) \hfill \\ 0 \hfill \\ \end{gathered} \right.$$

### Algorithm procedure

**Figure 9:**
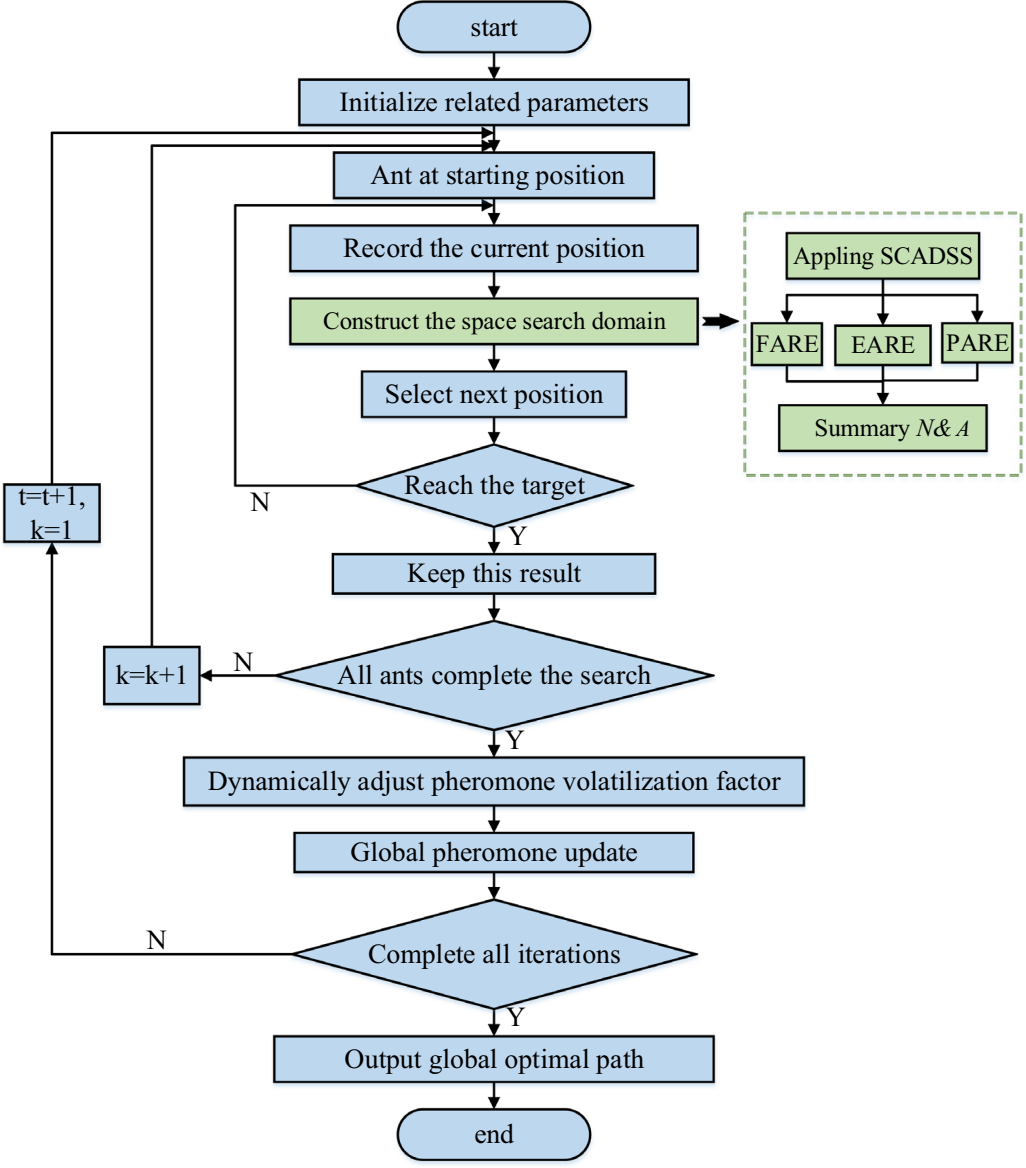
Algorithm procedure.

The improved algorithm procedure in this paper, as shown in Figure 9.

## Simulation experiment and result analysis

### Search strategy validation analysis

In the same 10*10*10 environment map, two groups of simulation experiments are performed with basic ACO. Each group of simulation experiment plans a shortest path from the same starting location to the same target location. The rationality and effectiveness of SCADSS are verified indirectly by the path planning results of simulation experiments.

**Figure 10:**
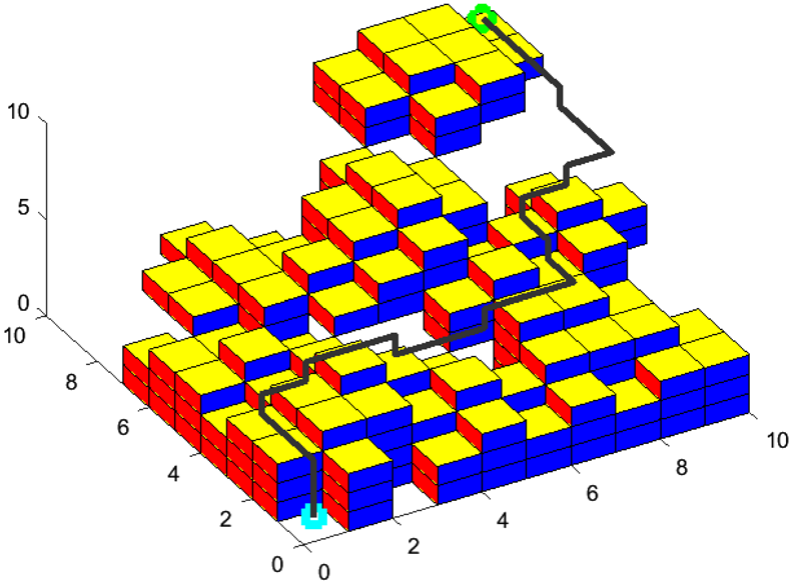
Path simulation of group-I.

**Figure 11:**
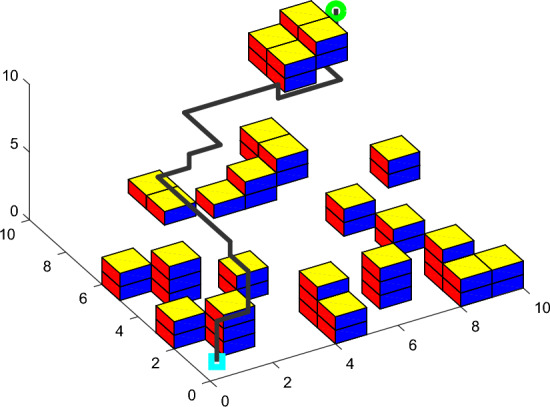
Path simulation of group-II.

Group-I of simulation experiment: implement the expansion object obstacle treatment and planar search strategy. Group- II of simulation experiment: implement spatial connectivity adjacent domain search strategy. Compare and verify the rationality and effectiveness of SCADSS. The experimental results are shown in Figs. 10, 11.

Analyzing the statistical data in Table 1, the implementation of both strategies meets the basic requirements of safe navigation of AUV in path planning. There is no significant difference in the length of experimental path between the two groups’ results. However, the experimental results of implementing SCADSS showed that the number of turning times decreased from 19 to 15, and the path tortuosity decreased by 21.05%. SCADSS avoids unnecessary obstacles in environment modeling and ensures the scale of traversable space for path search. So a large solution set space is created for the optimal solution, and the success rate of path search is increased by 3%. It can be proved that the SCADSS strategy has obvious rationality and superiority, and is more conducive to smooth path planning of AUV kinematic characteristics.

**Table 1:** Search strategy validation analysis.

Comparison index	Simulation experiment I	Simulation experiment II
Map scale	10*10*10	10*10*10
Start position	(1000,1000,1000)	(1000,1000,1000)
Target position	(1,1,1)	(1,1,1)
Proportion of actual obstacle area	25/1000	25/1000
Increase proportion of expansion obstacle	139/1000	0
$PL$	29.00	29.00
$P\theta$	1710.00	1350.00
Turning times	19	15
$fit$	1.18	1.03
Path search success rate	62%	65%

### Parameter optimization

In the experiment, the parameters of the ant colony optimization are initialized as follows: $T = 100$, $M = 50$, $\alpha = 1.5$, $\beta = 10$, $Q = 1$,$\rho_{1} = 0.3$.Optimize and rectify the introduced parameter $\gamma$,$\varepsilon$.The parameter options are: $\gamma \in \{ 1,2,3,4\}$, $\varepsilon \in \{ 1,2,3,4\}$, other parameters remain unchanged by default. After the optional parameters are combined and arranged. Under the condition that 10 * 10 * 10 map start and target positions are the same. Take the average value of 20 simulation experiments. The experimental results are shown in Table 2. Compared with the indicators of the results, the optimal values of the parameters designed in this paper are:$\gamma = 3$,$\varepsilon = 4$.

**Table 2:** Parameter optimization analysis.

$\gamma$	1	1	1	1	2	2	2	2
$\varepsilon$	1	2	3	4	1	2	3	4
$PL$	17.68	17.73	18.07	17.39	17.59	17.39	17.44	17.68
$P\theta$	298.56	267.76	228.03	258.03	237.77	218.295	251.33	270.80
$Pfit$	0.20	0.185	0.185	0.17	0.17	0.15	0.17	0.19
Turning times	6–8	5–6	5–5	5–6	4–5	5–6	6–7	5–7
$\gamma$	3	3	3	3	4	4	4	4
$\varepsilon$	1	2	3	4	1	2	3	4
$PL$	17.73	17.98	17.00	17.05	17.59	17.83	17.68	17.73
$P\theta$	237.77	205.53	138.03	115.53	157.50	231.06	218.30	228.03
$Pfit$	0.17	0.17	0.105	0.095	0.135	0.175	0.165	0.17
Turning times	4–7	4–5	2–5	2–4	2–4	5–7	5–5	4–5

### AUV path planning simulation experiment

To verify the effectiveness of the IACO algorithm proposed in this paper in robot 3D path planning. ACO and UACO^30^ are used as comparison algorithms. The simulation comparison test is conducted in 10 * 10 * 10 of the longitudinal obstacle dense environment map. The three algorithm parameter settings are shown in Table 3.

**Table 3:** Parameter settings.

$\alpha$	1.5	1.5	1.5
$\beta$	10	10	10
$Q$	1	1	1
$\rho$	0.5	$\rho (t + 1) = \max [0.95\rho (t),0.1]$	$\rho (t + 1) = \frac{1}{2\sqrt \pi }e^{{ - \frac{{(\rho (t) - \rho_{f} (t))^{2} }}{2}}}$

**Figure 12:**
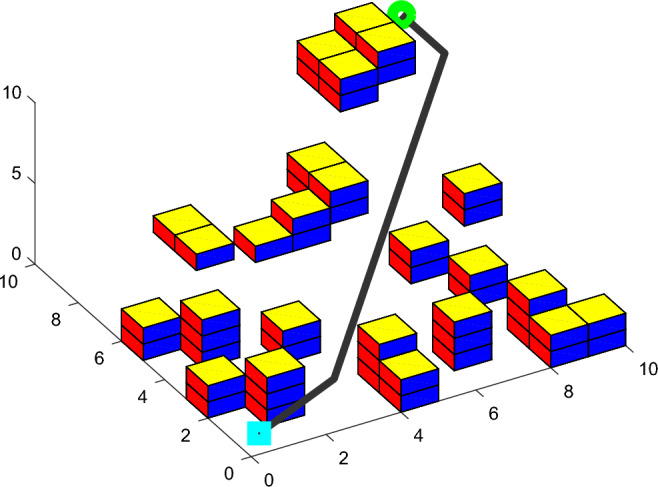
Optimal path simulation of I-ACO.

**Figure 13:**
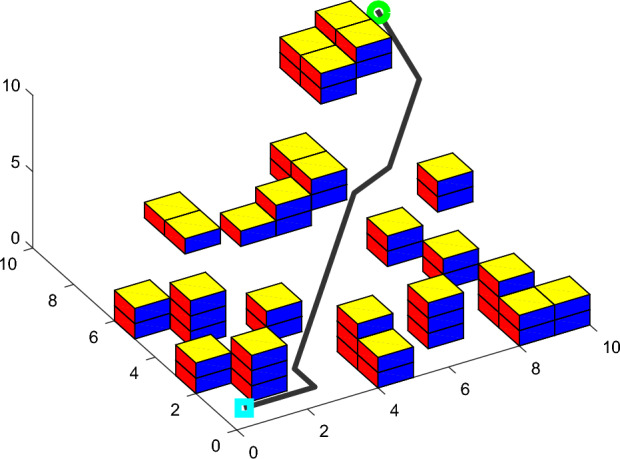
Optimal path simulation of U-ACO.

**Figure 14:**
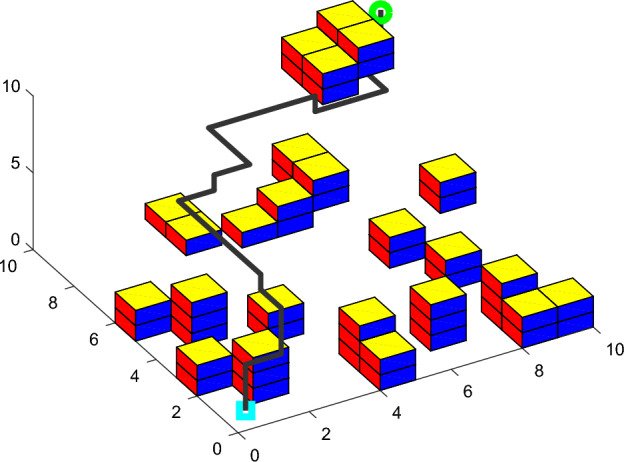
Optimal path simulation of ACO.

**Figure 15:**
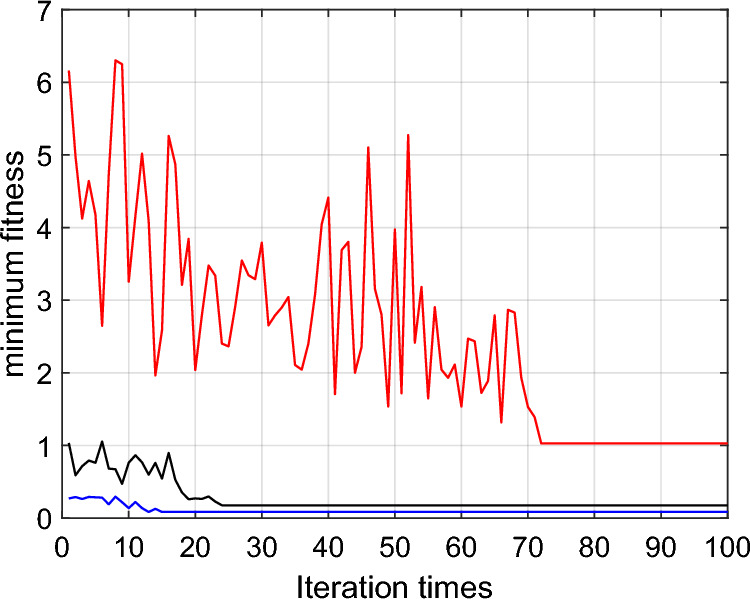
*fit* iteration curves.

In the 10 * 10 * 10 grid map, the optimal path simulation of the three algorithms are shown in Figures 12, 13, 14. The *fit* iteration curves are shown in Figure 15. The blue curve is the *fit* iteration result of IACO. The black curve is the *fit* iteration result of UACO. The red curve is the *fit* iteration result of ACO. The experimental results are shown in Table 4.

**Table 4:** Result analysis.

	I-ACO	U-ACO	ACO
$\min PL$	16.95	17.63	29.00
$\min P\theta$	90.00	254.00	1350.00
$\min fit$	0.08	0.18	1.03
Turning times	2	5	15
Best result algebra	13	24	72

Table 4 shows that the shortest path length obtained by IACO algorithm is 3.86% shorter than that obtained by UACO algorithm and 41.55% shorter than that obtained by ACO algorithm. In addition, compared with UACO and ACO algorithms, the turning times of the optimal path obtained by IACO algorithm are reduced by 60% and 86.67% respectively. The adaptability of the planned path obtained by IACO algorithm is optimal, balancing the requirements of path length and path transition. According to the iteration curve of the IACO algorithm, there are obvious advantages in the initial stage of iteration. Moreover, the optimization value decreases steadily, almost without sudden change and jump fluctuation, and the search speed is 45.83% and 81.94% higher than that of UACO and ACO respectively. Therefore, the IACO algorithm proposed in this paper has stronger optimization ability in AUV path planning.

## Conclusion

Considering the influence of connectivity between search locations on path security, the spatial connectivity adjacency search domain strategy is designed. On the premise of not reducing the free area in the space, the space security search of the path can be realized. SCADSS broadens the view of path search and is beneficial to the generation of smooth path. Combined with SCADSS, ACO algorithm is optimized to solve the problem of path length and path tortuosity of AUV in space obstacle dense environment. The local turn heuristic function is designed to improve the superiority of local direction selection. The global comprehensive guidance heuristic function and the improved distance heuristic function are designed to improve the ability of searching the equilibrium solution with the shortest path length and the least path tortuosity. Dynamic adjustment strategy of pheromone volatile factor improves the sensitivity of the algorithm to the solution quality. The simulation experiments select the most suitable algorithm parameters, verify the correctness of the spatial search strategy and the superiority and rapidity of the improved ACO algorithm.

### Supplementary Information

Supplementary Information.

## Data Availability

Because the data in the article relates to the research privacy of some authors, the data sets generated and/or analyzed in the current study are not publicly available but are available at the reasonable request of the corresponding authors.
