# Author Correction: 3D smooth path planning of AUV based on improved ant colony optimization considering heading switching pressure

**DOI:** 10.1038/s41598-023-41140-2

**Published:** 2023-08-28

**Authors:** Ronghua Meng, Aiwen Sun, Zhengjia Wu, Xuan Du, Yongdong Meng

**Affiliations:** 1 Hubei Key Laboratory of Construction and Management in Hydropower Engineering, China Three Gorges University, Yichang, 443002 Hubei China; 2 Hubei Key Laboratory of Hydroelectric Machinery Design and Maintenance, China Three Gorges University, Yichang, 443002 Hubei China; 3 Intelligent Manufacturing Innovation Technology Center, China Three Gorges University, Yichang, 443002 Hubei China; 4 School of Management, Jinan University, Guangzhou, 510632 Guangdong China

Correction to: *Scientific Reports* 10.1038/s41598-023-39346-5, published online 31 July 2023

The Data Availability section of the original version of this Article was incorrect.

“The datasets from the “Hubei Key Laboratory of Hydroelectric Machinery Design and Maintenance” but restrictions apply to the availability of these data, which were used under license for the current study, and so are not publicly available. Data are, however, available from the corresponding author upon reasonable request and with permission of Hubei Key Laboratory of Hydroelectric Machinery Design and Maintenance.”

now reads:

“Because the data in the article relates to the research privacy of some authors, the data sets generated and/or analyzed in the current study are not publicly available but are available at the reasonable request of the corresponding authors."

The original Article has been corrected.

