# Supplementary material for: 3D smooth path planning of AUV based on improved ant colony optimization considering heading switching pressure
Source: Sci Rep. 2023 Jul 31;13:12348. doi: 10.1038/s41598-023-39346-5 (PMC10390500; doi:10.1038/s41598-023-39346-5)
Supplement: Supplementary file 1 — Supplementary Information. [file 41598_2023_39346_MOESM1_ESM.zip › raw data/Instructions for use.docx]

| **Program file name** | **instruction** |
| --- | --- |
| field.mat | environmental data |
| xiuzheng27_yubei.m | Implementation code of Spatial Connectivity Adjacent Domain Search Strategy (SCADSS) |
| IACO.m | Improved intelligent algorithm code |
| diedaitu.m | Data visualization, code of iteration diagram and simulation diagram |
| matlab_0.08_90.mat | All the test data of the IACO algorithm presented in this paper |

**The operating environment** is "Matlab R2017b".
